# Phenotypic characterisation and linkage mapping of domestication syndrome traits in yellow lupin (*Lupinus luteus* L.)

**DOI:** 10.1007/s00122-020-03650-9

**Published:** 2020-07-18

**Authors:** Muhammad Munir Iqbal, William Erskine, Jens D. Berger, Matthew N. Nelson

**Affiliations:** 1 School of Agriculture and Environment, The University of Western Australia, Perth, WA 6009 Australia; 2 Centre for Plant Genetics and Breeding, The University of Western Australia, Perth, WA 6009 Australia; 3 The UWA Institute of Agriculture, The University of Western Australia, Perth, WA 6009 Australia; 4 CSIRO Agriculture and Food, Floreat, WA 6014 Australia; 5 Royal Botanic Gardens, Kew, Wakehurst Place Ardingly, West Sussex, RH17 6TN UK

## Abstract

The transformation of wild plants into domesticated crops usually modifies a common set of characters referred to as ‘domestication syndrome’ traits such as the loss of pod shattering/seed dehiscence, loss of seed dormancy, reduced anti-nutritional compounds and changes in growth habit, phenology, flower and seed colour. Understanding the genetic control of domestication syndrome traits facilitates the efficient transfer of useful traits from wild progenitors into crops through crossing and selection. Domesticated forms of yellow lupin (*Lupinus luteus* L.) possess many domestication syndrome traits, while their genetic control remains a mystery. This study aimed to reveal the genetic control of yellow lupin domestication traits. This involved phenotypic characterisation of those traits, defining the genomic regions controlling domestication traits on a linkage map and performing a comparative genomic analysis of yellow lupin with its better-understood relatives, narrow-leafed lupin (*L. angustifolius* L.) and white lupin (*L. albus* L.). We phenotyped an F_9_ recombinant inbred line (RIL) population of a wide cross between Wodjil (domesticated) × P28213 (wild). Vernalisation responsiveness, alkaloid content, flower and seed colour in yellow lupin were each found to be controlled by single loci on linkage groups YL-21, YL-06, YL-03 and YL-38, respectively. Aligning the genomes of yellow with narrow-leafed lupin and white lupin revealed well-conserved synteny between these sister species (76% and 71%, respectively). This genomic comparison revealed that one of the key domestication traits, vernalisation-responsive flowering, mapped to a region of conserved synteny with the vernalisation-responsive flowering time *Ku* locus of narrow-leafed lupin, which has previously been shown to be controlled by an *FT* homologue. In contrast, the loci controlling alkaloid content were each found at non-syntenic regions among the three species. This provides a first glimpse into the molecular control of flowering time in yellow lupin and demonstrates both the power and the limitation of synteny as a tool for gene discovery in lupins.

**Electronic supplementary material:**

The online version of this article (10.1007/s00122-020-03650-9) contains supplementary material, which is available to authorised users.

## Introduction

Plant domestication refers to the transformation of wild progenitor plants into crops meeting human needs that are well adapted to agricultural production (Diamond 2002). Domestication of plant species occurred in different parts of the world over various time periods with the greatest intensity between 3000 and 12,000 years ago (Diamond 2002). This resulted in the genetic and morphological differences between domesticated plants and their wild progenitors for a common set of traits in many crop species referred to as the ‘domestication syndrome’ (Hammer 1984). The domestication syndrome generally includes loss of seed dormancy, acquisition of seed indehiscence, a more upright and apically dominant growth habit, reduction in anti-nutritional components of seeds and enhanced plant vigour (Diamond 1998; Hammer 1984; Meyer et al. 2012). Early phenology can also be considered a domestication trait for crops such as lupins, as it allows them to complete their life cycle before the onset of terminal drought in Mediterranean-type environments and before autumn in cool temperate environments. This earliness enabled the cultivation of lupins in new climates in Northern Europe and then Australia (Taylor et al. 2020).

Yellow lupin (*Lupinus luteus* L., 2*n* = 52) is a winter-annual legume from the Mediterranean Basin and Iberian Peninsula (Gladstones 1998). Unlike other major legume and cereal crops of economic importance such as wheat (*Triticum aestivum* L.), rice (*Oryza sativa* L.), corn (*Zea mays* L.), and chickpea (*Cicer arietinum* L.), yellow lupin has a very recent, short and fragmented domestication history (Berger et al. 2013; Cowling et al. 1998; Hondelmann 1984). Because modern lupin breeding is ca. 100 years old and has been well documented (von Sengbusch 1930; von Sengbusch and Zimmermann 1937), the timing and severity of domestication bottlenecks is known, in contrast to the situation in the Neolithic founder crops listed previously. For example, von Sengbusch screened > 4 million *Lupinus angustifolius* L. plants for indehiscence, selecting 140,000 candidates for further development (von Sengbusch 1930; von Sengbusch and Zimmermann 1937). Subsequently, Gladstones reselected on pod shattering (Gladstones 1967), which added a further population bottleneck. Because the reduced genetic diversity associated with these bottlenecks is likely to limit adaptation and future cultivar development, it is important to have a good genetic and genomic understanding of the domestication traits in yellow lupin (Iqbal et al. 2020). If we understand which loci are essential in maintaining a domesticated crop, then by extension we also know where it is safe to allow more genomic diversity to be brought in from wild types of yellow lupin.

While yellow lupin and narrow-leafed lupin share a similar history of domestication, the genetics of key domestication traits is much better understood in narrow-leafed lupin (Cowling et al. 1998; Nelson et al. 2010a; Plewiński et al. 2019). Domestication traits in narrow-leafed lupin are typically controlled by recessive alleles at one or two loci: low alkaloid (*iucundus*), water-permeable seeds (*mollis*), seed indehiscence (*tardus* and *lentus*), and flower, seed and hypocotyl colour (*leucospermus*). Early flowering in narrow-leafed lupin is controlled by an *FT* homologue present at the dominant locus *Ku*, which removes the requirement of vernalisation to induce flowering (Nelson et al. 2017; Taylor et al. 2019). Early flowering is a crucial adaptive trait for short season Mediterranean environments where it enables drought escape and full seed maturity at harvest (Berger and Ludwig 2014; Gladstones 1977).

In yellow lupin, similar trait contrasts are found that distinguish cultivated from wild forms, but the genetic control of these key domestication traits is unknown (Gladstones 1970). For example, wild yellow lupin germplasm exhibits pod dehiscence, hard seededness (physical seed dormancy), rosette growth habit and is highly vernalisation-responsive (requiring cool temperatures to induce flowering) (Cowling et al. 1998; Gladstones 1998; Zohary et al. 2012). The objective of this study was to understand the genetics of these domestication traits in yellow lupin and identify the loci controlling these traits on a newly developed linkage map of yellow lupin (Iqbal et al. 2019). A comparison of the yellow lupin genome to its better characterised and resourced sister species, narrow-leafed lupin and white lupin (*L. albus* L.), provided potential candidate genes for future molecular analysis of domestication genes in yellow lupin.

## Materials and methods

### Plant material

A total of 156 yellow lupin (*Lupinus luteus* L.) F_9_ recombinant inbred lines (RILs) of a bi-parental cross of domesticated (Wodjil cultivar) × wild (Australian Lupin Collection accession P28213 originating in the Azores, 37° 44′ 28.5″ N, 25° 40′ 33.0″ W), along with parents were grown in experimental field area at CSIRO Floreat (31° 57′ 00.7″ S, 115° 47′ 18.8″ E), Perth, Australia. This population was developed and provided by the Department of Primary Industries and Rural Development (South Perth, Australia). All seeds were scarified to ensure all seeds imbibed at the same time irrespective of hard/soft seed status. Ambient temperature was recorded using in-field thermal monitors.

### Experimental design

All the genotypes were grown in a split-plot design under two vernalisation treatments, applied to contiguous regions of a single field: (a) vernalised: imbibed seeds in Jiffy pots were given vernalisation treatment at 8 °C for 21 days starting 21st May 2013 and transferred to field on 11th June, 2013; (b) non-vernalised: directly sown into field without any vernalisation treatment on 7th–8th June, 2013. There were four replications of genotypes, with the two parents (Wodjil and P28213) replicated eight times in each main plot. The plant × plant distance was 10 cm within rows and a row-to-row distance of 25 cm. DiGGer was employed to randomise the design (Coombes 2009).

### Phenotyping

The following traits were measured in the experimental RIL population:

The response to vernalisation for each genotype was measured as the difference in time to 50% flower (days) between ± vernalisation treatments. The time to 50% flowering (days) was recorded from transplanting date (for vernalisation treatment) and emergence date (for non-vernalisation treatment) to the day when 50% of the plants in a plot flowered (first petals appeared). The RIL population was also categorised as early and late flowering based on a cut-off value of average difference between both treatments, and these categories were used later for linkage mapping. The time to maturity (days) was recorded when all pods were brown and seeds hard. The data for time to 50% flowering and time to maturity were used to calculate the length of reproductive phase (days) as follows.

The length of reproductive phase (days) = time to maturity (days) − time to 50% flowering (days).

Flower colour was recorded as bright yellow (P28213) or light yellow (Wodjil). Growth habit was recorded as upright (Wodjil) or rosette (P28213). Growth habit was assessed based on the presence/absence of main stem and point of emergence of lateral branches during first 2–3 weeks of growth period. The upright plants had a well-established main stem and lateral branches originating from axillary buds on main stem. While rosette plants did not have a well-established main stem and lateral branches originated directly from the axillary meristems near root area, which made them lay on the ground because of absence of any support.

Leaves were assayed for presence or absence of alkaloids. Leaves were plucked off at the petiole and petioles dabbed on Dragendorff paper (Sreevidya and Mehrotra 2003). The leaf sap of alkaloid-containing plants changed the colour of Dragendorff paper to red, while alkaloid-free RILs did not change colour. Leaf alkaloid content serves as a proxy for seed content as found by Phan et al. (2007).

Two-character states were recorded for pod dehiscence upon ripening: dehiscent and indehiscent. This variable was recorded twice: First at physiological maturity and then after drying in bags. As some lines did not show clear-cut dehiscent or indehiscent phenotypes, a cut-off value of 50% was used and lines with more than 50% indehiscent pods were categorised as indehiscent and vice versa. Seed testa colour was recorded as black with white marbling or white.

The hardness/softness of the seed (seed-coat permeability) was recorded after harvest by an imbibition test. The assay was conducted approximately five months after harvest to avoid potential confounding with primary dormancy in newly harvested seeds. A total of 20–25 seeds from each RIL were placed in a Petri dish upon moist filter paper. After 24 h, lines with swollen seeds were recorded as soft-seeded and the unswollen lines as hard-seeded. For confirmation, the dishes were maintained at room temperature for 5–7 days to test seed germination percentage. The lines were then classified as soft- or hard-seeded at a cut-off value of 75% of seeds in a Petri plate germinated or ungerminated, respectively.

### Phenotypic data analysis

The segregation ratios of qualitative data were tested using Chi square (*χ*^2^) (Levine et al. 2001) in order to investigate the possible number of genes controlling a trait. Traits were first tested for the single gene hypothesis, where the Mendelian expectation for a RIL population is 1:1. The segregation values of those traits were then tested for epistasis ratios 9:7, 13:3, 12:4/3:1 and 15:1 ratios in order to find any gene interactions, especially where a trait was controlled by more than one gene. Quantitative traits such as time to flowering, time to maturity and length of reproductive phase were analysed under both treatments using split-plot analysis of variance (ANOVA) in the 17th edition of GenStat (VSN International, UK).

### Genotyping and linkage mapping

Marker genotyping and linkage mapping data were used from recently published linkage map of yellow lupin (Iqbal et al. 2019). A total of 2450 single nucleotide polymorphism (SNP) and presence/absence variation (PAV) markers along with seven phenotypic traits, viz. vernalisation response, plant growth habit, flower colour, seed colour, alkaloid content, pod dehiscence and seed permeability, were mapped using MultiPoint3.3 software (Mester et al. 2003). The quantitative trait loci (QTL) analysis for vernalisation response was conducted using 17th edition of GenStat. QTL analysis involved four steps: (1) identification of the most appropriate model; (2) simple interval mapping (SIM) in which main effect QTLs are calculated against the default threshold − log10(P) = 3.82 and QTL × E interactions estimated; (3) composite interval mapping (CIM) using SIM-derived QTLs as co-factors; and (4) A final scan, all candidate QTLs are compared and effect of each QTL calculated.

### Comparison of yellow lupin and narrow-leafed lupin genomes

The comparative genome analysis was achieved by aligning the yellow lupin sequence tags of 2458 SNP and PAV markers (90 nt for GBS markers; 69 nt for DArTseq markers) to the Tanjil reference genome sequence of narrow-leafed lupin (Hane et al. 2017) and white lupin AMIGA reference genome (Hufnagel et al. 2020). The analysis was carried out using blast-2.9.0 + (http://ftp.ncbi.nlm.nih.gov/blast/executables/blast+/2.9.0/) employing the options -task blastn-short -evalue 1e^−5^ -max_target_seqs 3. The most significant match was used except when the second or third match fitted established patterns of synteny. Results were visualised by GridMap 3.0 (http://cbr.jic.ac.uk/dicks/software/Grid_Map/). Synteny between the yellow lupin vs narrow-leafed lupin and white lupin genomes was visualised using Circos (Krzywinski et al. 2009).

## Results

### Phenotyping and segregation ratios of domestication traits

**Fig. 1:**
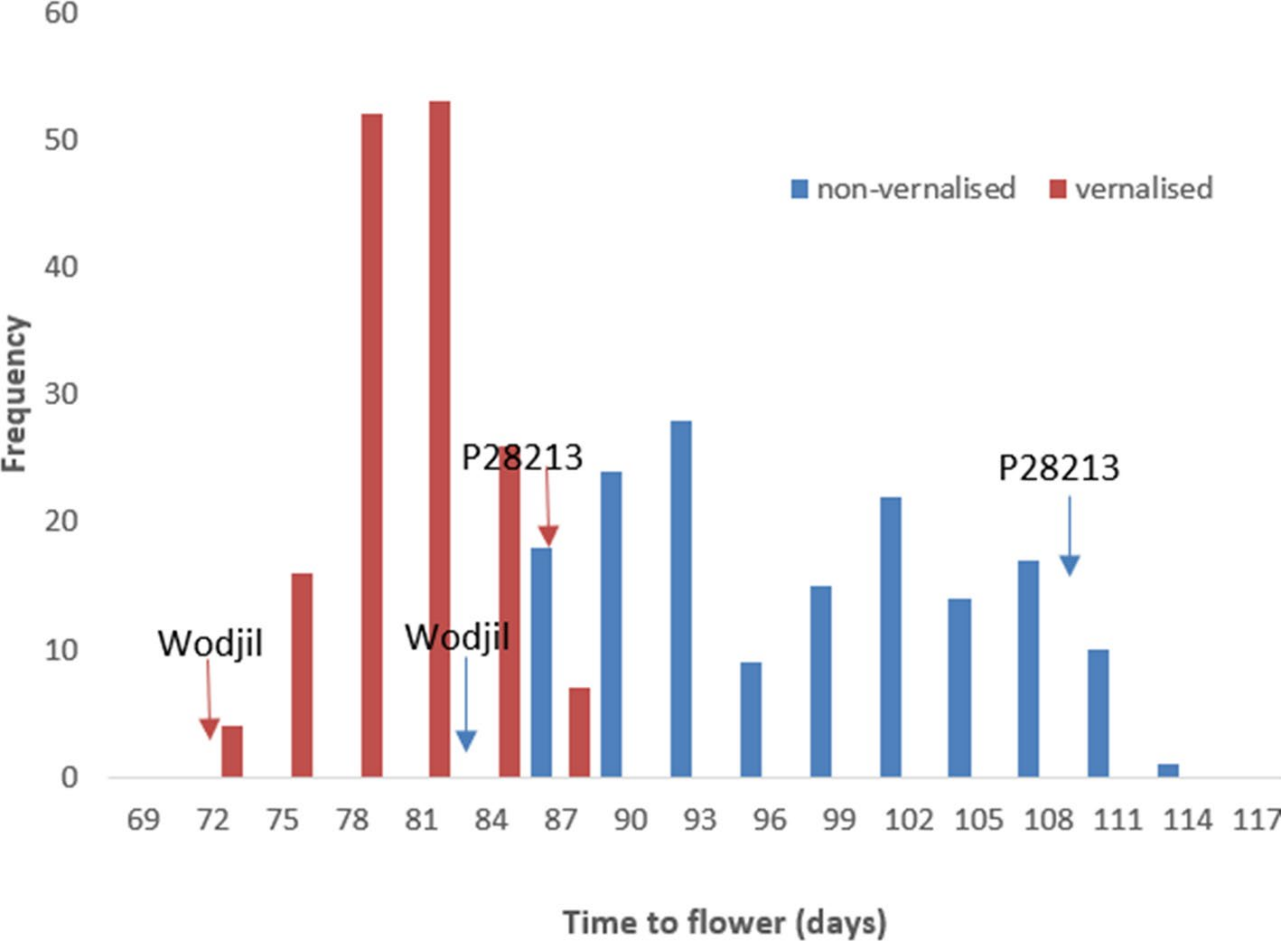
Time to flower (days) under ± vernalisation treatments in a yellow lupin recombinant inbred line population (*n* = 156) developed from a cross between cultivar Wodjil and wild accession P28213. Arrows indicate the flowering times of the two parents

**Fig. 2:**
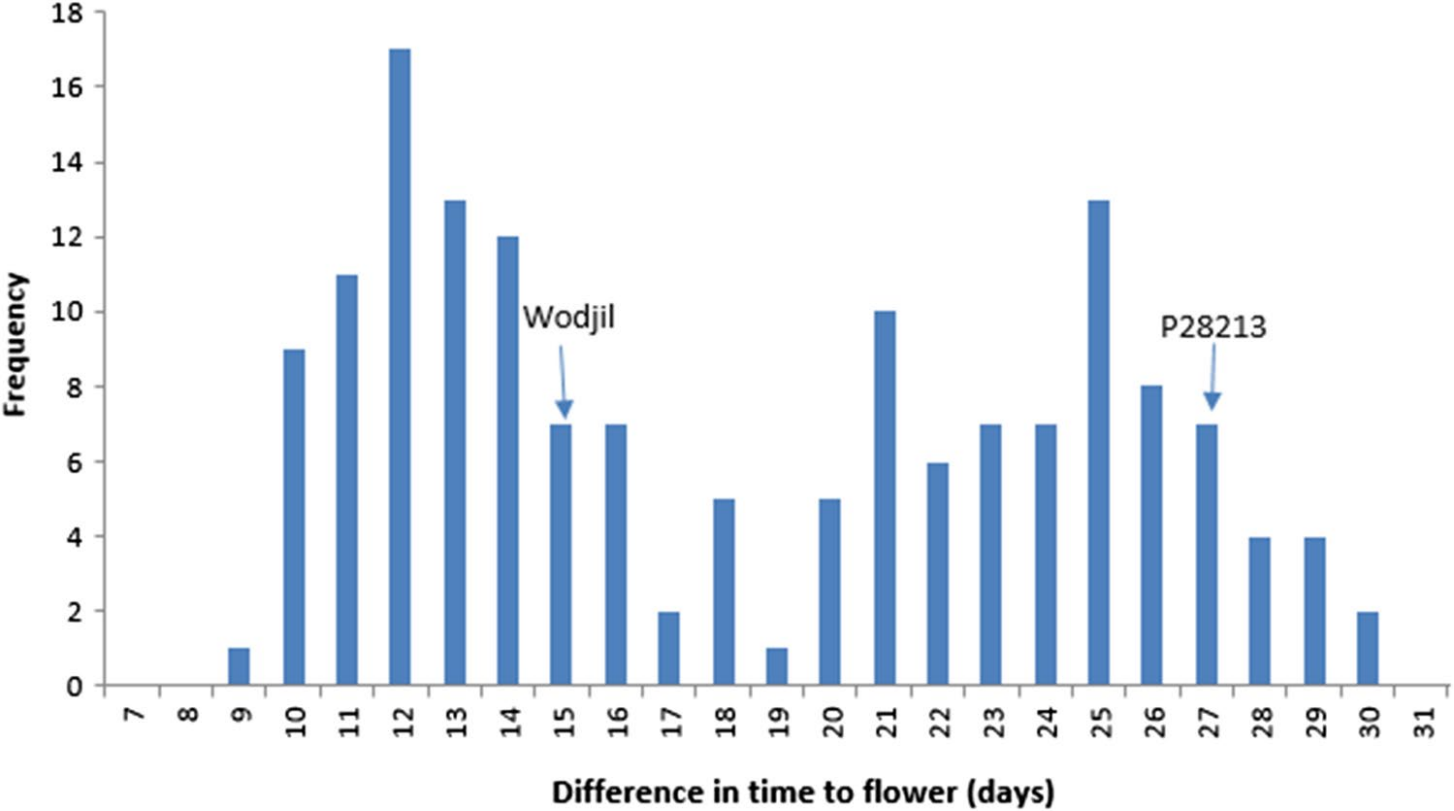
Response to vernalisation expressed as difference in time to flowering (days) between ± vernalisation treatments in a yellow lupin recombinant inbred line population (*n* = 156) developed from a cross between cultivar Wodjil and wild accession P28213. Arrows indicate the vernalisation responses of the two parents

ANOVA revealed significant variation in time to flower among genotypes in the RIL population with largest effect due to ± vernalisation treatment, followed by genotypic effect and interaction effect (Table 1). This hierarchy of effects could be observed as a clear positive correlation between time to flowering in vernalised and non-vernalised RILs, but with noise around the trend indicating that some RIL responded more strongly to vernalisation than others (Fig. 1). The range of time to flower under vernalised treatment was between 70 and 90 days, while the same population took 84–115 days under non-vernalised treatment (Fig. 1). Vernalisation significantly accelerated flowering by 9–31 days (Fig. 2). The bimodal distribution suggested single-gene control for vernalisation response, which was confirmed by a Chi-square analysis (*χ*^2^ *P* < 0.05) (Table 2, Table S1). The segregation ratio for vernalisation response also fitted the ratio of 9:7, indicating genetic control by duplicate recessive epistatic effect of two genes (Table S2).

**Table 1:** Table of means squares (m.s.) from ANOVA for genotype (genotypic mean square—G m.s.), treatment (expressed as vernalisation mean square—Vern m.s.) and genotype × treatment interaction (expressed as genotype × vern interaction mean squares—G × Vern m.s.) for time to flower (days), time to maturity (days) and time of reproductive phase (days) under ± vernalisation treatments in an F_9_ recombinant inbred line population of yellow lupin (*n* = 156)

	G m.s.	Vern m.s.	G x Vern m.s.
Time to flower	193.6***	989474.3***	77.4***
Time to maturity	9.7***	46144.3***	9.5***
Length reproductive phase	206.4***	7275.9**	82.6***
d.f.	155	1	155

*p* < 0.05 (*), *p* < 0.01 (**), *p* < 0.001 (***)

**Table 2:** Chi-square (*χ*^2^) *P* values for domestication traits of yellow lupin segregating for domesticated alleles (Wodjil parent) and wild alleles (P28213) in an F_9_ recombinant inbred line population (*n* = 156) of yellow lupin. Chi-square (1 gene) results are based on a by one-gene model (1:1 ratio)

	Wodjil (allele counts)	P28213 (allele counts)	*χ*^2^ *P* value (1 gene)	Best fit model
Vernalisation response	Responsive (81)	Unresponsive (66)	0.182NS	One gene
Flower colour	Light yellow (69)	Bright yellow (85)	0.197NS	One gene
Growth habit	Upright (118)	Rosette (40)	5.45E−10***	Two genes
Alkaloid content	Low alkaloid (54)	Alkaloid containing (97)	0.000***	One or two genes
Dehiscence	Indehiscent (47)	Dehiscent (110)	4.96E−07***	Two genes
Hard/soft seed	Soft (123)	Hard (35)	2.54E−12***	Two genes
Seed colour	White (74)	Black (82)	0.521NS	One gene

*P* < 0.001***, *P* < 0.05*, *P* ≥ 0.05 non-significant

Early flowering genotypes had a longer reproductive phase than late flowering genotypes which had a reduced reproductive phase (Supplementary Figure 1a). Overall, the entire RIL population completed its growth cycle at around the same time (Supplementary Figure 1b).

**Fig. 3:**
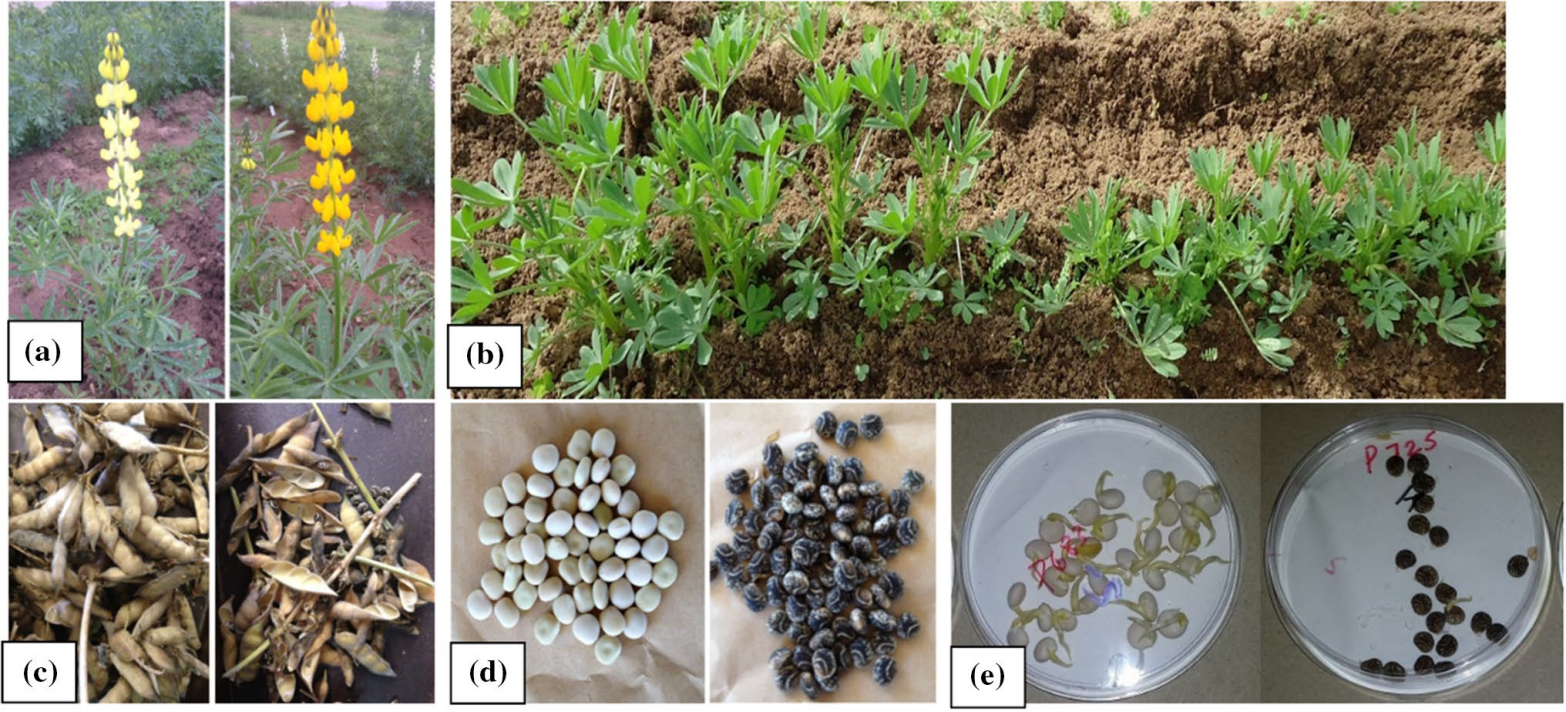
Domestication traits recorded in the RIL population (**a**) flower colour, i.e. light yellow (Wodjil) and bright yellow (P28213), (**b**) growth habit, i.e. upright (Wodjil) and rosette (P28213), (**c**) pod-dehiscence type, i.e. indehiscent (Wodjil) and dehiscent (P28213), (**d**) seed colour, i.e. white (Wodjil) and black with white marbling (P28213), (**e**) seed permeability, i.e. soft seed (Wodjil) and hard seed (P28213) (colour figure online)

The parents P28213 and Wodjil expressed two distinct flower colours (bright yellow and light yellow, respectively) (Fig. 3a) and the trait segregated in the RIL population in the 1:1 ratio of single-gene control (Table 2). This trait also fitted to a 9:7 ratio, suggesting control by duplicate recessive epistasis (Table S2).

The RIL population showed two distinct growth habits: rosette (P28213) and upright (Wodjil) (Fig. 3b). Chi-square analysis indicated that growth habit did not fit the Mendelian ratio of 1:1 indicating more than one gene governing this trait in yellow lupin (Table 2). The observed counts for growth habit fitted dominant epistasis ratios of 12:4 = 3:1 and also 13:3 indicating that dominant suppression by two genes is governing this trait in yellow lupin (Table S2).

The alkaloid assay showed that the wild parent P28213 as high alkaloid, while the domesticated parent was low alkaloid. This trait did not segregate according to a single gene (1:1) model (Table 2), but instead was found fit the model for duplicate recessive epistasis of 9:7 (Table S2).

The parents expressed contrasting dehiscence habits with the wild P28213 dehiscent and domesticated Wodjil indehiscent (Fig. 3c). This trait did not segregated in a 1:1 ratio expected for the one-gene control (Table 2). Instead, the segregation values for dehiscence were found to be goodness of fit to dominant epistasis ratios of 12:4 = 3:1 indicating that dominant alleles of two genes controlling this trait in yellow lupin (Table S2).

The RIL population showed very clear segregation of parental seed colour trait (Fig. 3d) in expected 1:1 ratio for a single gene (Table 2). On the other hand, segregation of seed colour was also found to be a goodness of fit for 9:7 ratio indicating duplicate recessive epistasis gene action (Table S2).

The screening of RIL population for seed permeability showed two categories, viz. hard-seeded and soft-seeded, while some lines showed an ambiguous response being semi-hard or semi-soft-seeded. The wild parent P28213 showed semi-hard characteristics (~ 50% of the seeds under seed permeability test showed no germination, while others germinated), while domesticated parent Wodjil was clearly soft-seeded (all seeds germinated). Based on the categorisation of ≥ 50% seeds in a line, the segregation ratio indicated that that seed permeability in yellow lupin is not controlled by a single gene (Table 2). The segregation values of seed permeability were found to fit the epistasis ratios of 12:4 = 3:1 and 13:3 indicating that dominant suppression by two different genes controlling the trait in yellow lupin (Table S2). This trait was also considered as a continuous variable in terms of  % seeds germinated in a Petri dish in order to find QTLs associated with this trait, but with no success (data not presented) (Fig. 3e).

### Linkage mapping of domestication traits

**Fig. 4:**
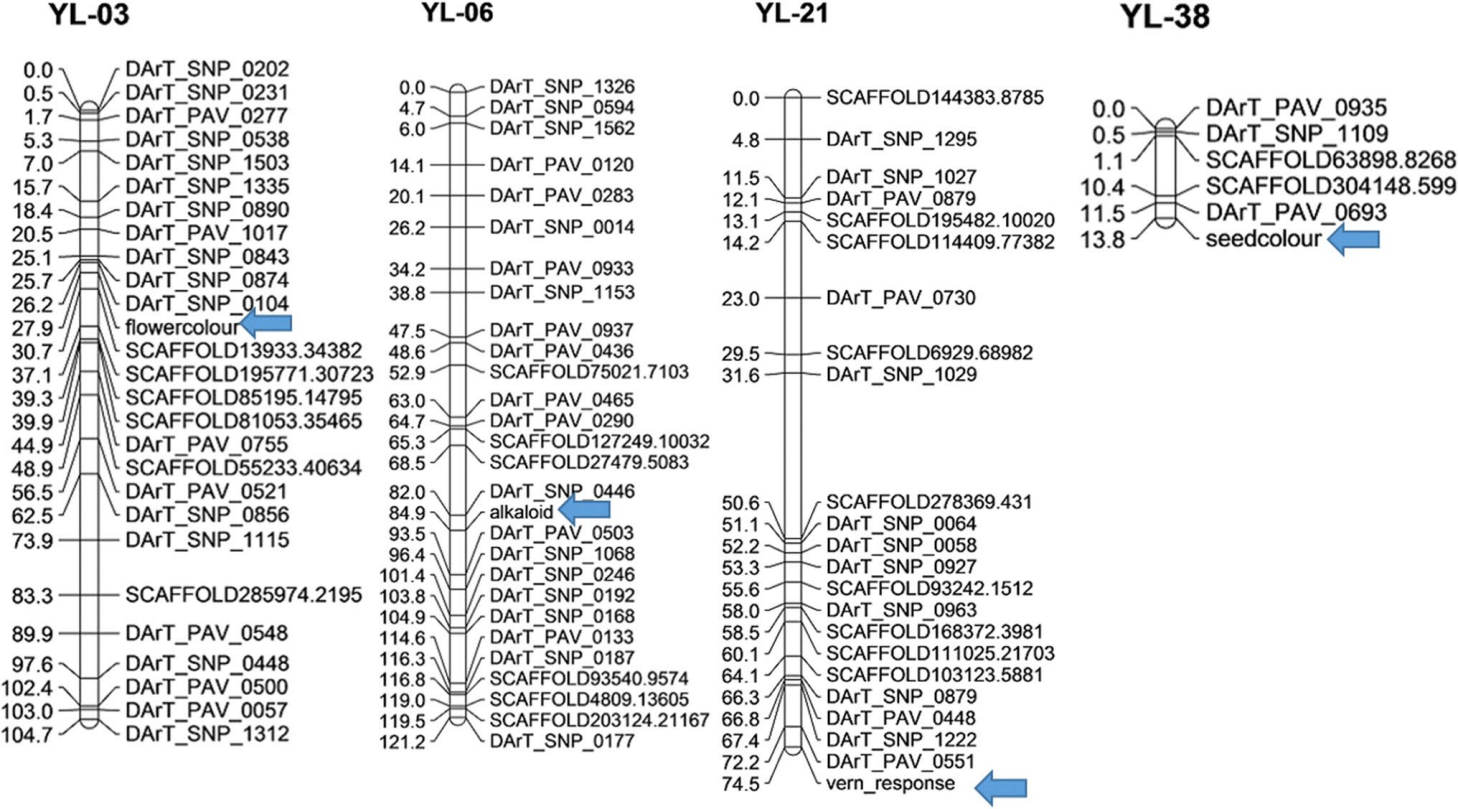
The locations of genomic regions controlling vernalisation response, flower colour, alkaloid content and seed colour on a yellow lupin linkage map reported by Iqbal et al. (2019). Arrows indicate the location of domestication traits

The six domestication traits scored according to parental genotypes were incorporated into the linkage map of yellow lupin (Iqbal et al. (2019). This approach successfully mapped flower colour to linkage group YL-03, alkaloid content to YL-06, vernalisation response to YL-21, and seed colour to YL-38 (Fig. 4), supporting single-gene control for these domestication traits (Table 2). The region of YL-06 where alkaloid content was mapped was significantly skewed towards the wild, bitter parent explaining the failure of alkaloid content to follow the expected Mendelian segregation ratios for a single gene trait (Table 2).

Growth habit, seed dehiscence and hard/soft seededness could not be mapped to linkage groups, consistent with their control by more than one locus (Table 2, Table S2).

### QTL analysis for vernalisation response

In the F_9_ RIL population, one significant QTL was found to control vernalisation response. This QTL mapped to linkage group YL-21, adjacent to the Mendelian vernalisation response locus (Fig. 4, Table 3). This locus explained 83% of total phenotypic variance, confirming the single gene model of its genetic control. No significant QTLs were found for time to maturity and reproductive phase.

**Table 3:** Summary of significant (*P* < 0.001) quantitative trait loci (QTL) positions controlling vernalisation response, total effect, additive effect, P value and LOD or − log (10) scores

Trait	Marker	Position chr# (cM)	Total effect (percent explained variance-PEV)	Additive effect	*P* value	− log(10) against threshold value = 3.84
Vernalisation response	vern_response	YL-21 (74.5)	83%	5.676	< 0.001	61.0

### Comparison of yellow and narrow-leafed lupin genomes

**Fig. 5:**
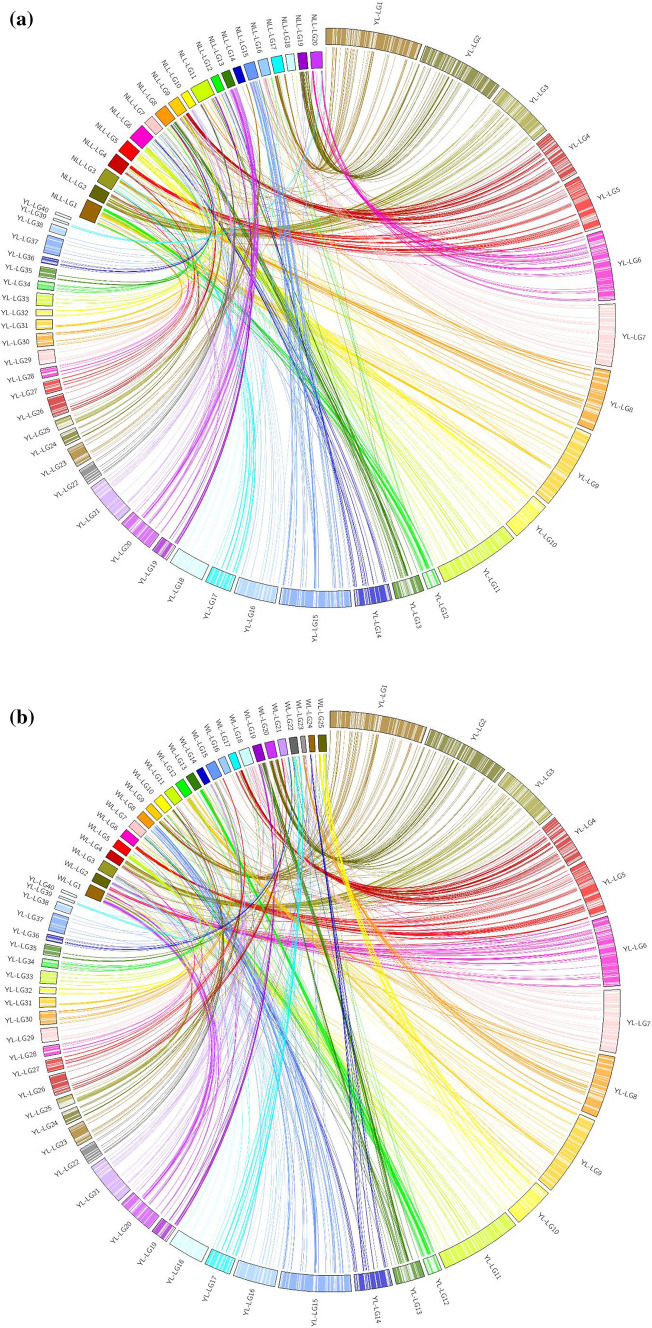
Global distribution of synteny between 40 linkage groups of yellow lupin (YL-01 to YL-40), 20 chromosomes of narrow-leafed lupin (NLL-01 to NLL-20) and 25 chromosomes of white lupin (WL-01 to WL-25). Loci showing homology between the two genomes at *P* < 1e^−5^ significance threshold are indicated by coloured lines from reference (yellow lupin) species linkage groups to target species; (** a**) narrow-leafed lupin and (**b**) white lupin chromosomes (colour figure online)

A total of 1874 (76%) and 1757 (71%) yellow lupin markers found significant (*P* < 1e^−5^) matches in the narrow-leafed lupin genome and white lupin genome, respectively (Table 4, Table S3). There was a high degree of variability in the extent of collinearity among the three genomes. Some yellow lupin groups aligned with narrow-leafed lupin and white lupin groups along most of their length (e.g. YL-01/NLL-17/WL-09; YL-07/NLL-16/WL-18; YL-23/NLL-14/WL-11). Some of yellow lupin groups aligned along most of their lengths to either of narrow-leafed lupin or white lupin but not with both (e.g. YL-04/WL-17; YL-21/NLL-10). Some apparent inversions within chromosomes were also observed (e.g. YL-02/NLL-19/Wl-20; YL-03/NLL-02/WL-04; Yl-12/WL-13 and YL-10/NLL-05/WL-25) (Fig. 5a, b). Some narrow-leafed lupin groups align to multiple yellow lupin groups (e.g. NLL-03/YL-08 and YL-17; NLL-04/YL-05, YL-24 and YL-27; NLL-06/YL-11, YL-14 and YL-16; NLL-08/YL-13, YL-26, YL-29, YL-34, YL-36 and YL-37; NLL-09/YL-03, YL-04, and YL-25). Similarly, some white groups align to multiple yellow lupin groups (e.g. WL-01/YL-10, YL-20, YL-25, YL-32, YL-40; WL-05/YL-11, YL-16; WL-10/YL-08, YL-22, YL-34; Wl30/YL-12, YL-30). Parts of narrow-leafed lupin and white lupin genomes did not appear to have clearly equivalent regions in yellow lupin (e.g. NLL-18; WL-16) (Fig. 5a, b).

**Table 4:** Numbers of yellow lupin loci with BLASTn (*P* < 1e^−5^) primary correspondences in twenty narrow-leafed lupin chromosomes (NLL-01–NLL-20) and twenty-five white lupin chromosomes (WL-01–WL-25)

Narrow-leafed lupin chromosome	Primary correspondences	White lupin chromosome	Primary correspondences
NLL-01	173	WL-01	130
NLL-02	160	WL-02	73
NLL-03	99	WL-03	68
NLL-04	143	WL-04	143
NLL-05	64	WL-05	101
NLL-06	141	WL-06	87
NLL-07	74	WL-07	36
NLL-08	143	WL-08	88
NLL-09	190	WL-09	69
NLL-10	39	WL-10	56
NLL-11	93	WL-11	24
NLL-12	38	WL-12	83
NLL-13	61	WL-13	82
NLL-14	22	WL-14	37
NLL-15	95	WL-15	41
NLL-16	49	WL-16	2
NLL-17	84	WL-17	129
NLL-18	11	WL-18	52
NLL-19	122	WL-19	117
NLL-20	73	WL-20	109
Total	1874	WL-21	39
		WL-22	41
		WL-23	59
		WL-24	29
		WL-25	62
		Total	1757

**Fig. 6:**
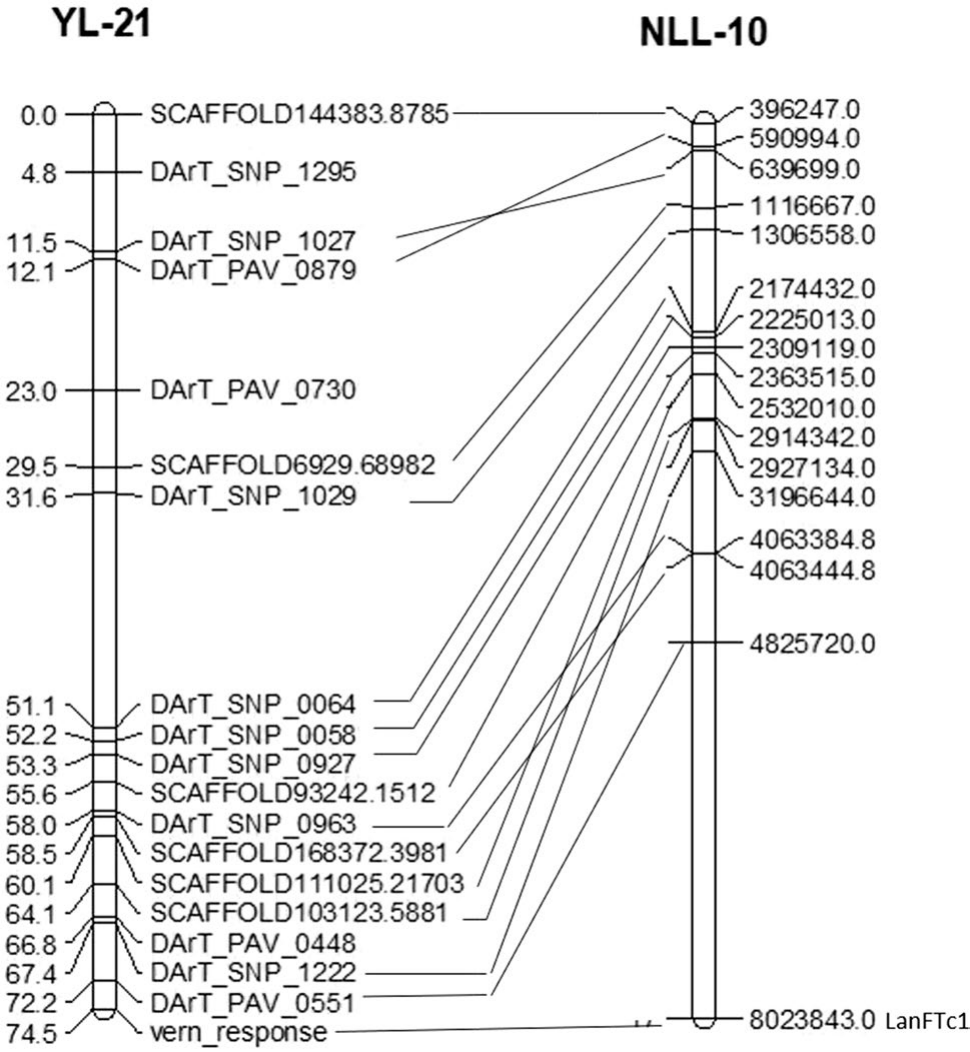
*Lupinus luteus* linkage group YL-21 showing conserved locus order with the upper section of *L. angustifolius* chromosome NLL-10. The vernalisation response locus is located at the bottom of YL-21. Names of loci showing conserved order are shown next to YL-21, and distances are shown in Kosambi centiMorgans (cM). The start nucleotide position of the syntenic match in *L. angustifolius* pseudomolecule NLL-10 is shown to the right of the pseudomolecule

Strikingly, alignment of the genomes revealed conserved positions for vernalisation response loci in yellow lupin and narrow-leafed lupin (the *Ku* locus) suggesting common genetic control (Fig. 6). As expected, the distinct flower and seed colour loci in yellow lupin were not syntenic with the combined flower/seed locus (*leucospermus*) in narrow-leafed lupin or white lupin. Interestingly, the alkaloid content locus mapped to a location distinct from the low alkaloid *iucundus* locus of narrow-leafed lupin and the *pauper* locus of white lupin, suggesting the low alkaloid mutations in these three species are different (data not presented).

## Discussion

This is the first genetic analysis of domestication traits in yellow lupin. We found that the domestication traits vernalisation response, alkaloid content, flower and seed colour are controlled by single genes (Table 2; Table S2), which were positioned on the genetic map of yellow lupin (Fig. 4; Table S3). This study also provided clear evidence of dominance suppression epistasis (Pooni and Treharne 1994) governing the genetic control of plant growth habit, seed permeability and pod dehiscence in yellow lupin. A genome comparison among yellow vs narrow-leafed lupins and white lupins showed well-conserved synteny between these sister species despite their difference in chromosome numbers. The linkage group containing vernalisation-responsive flowering time locus in yellow lupin showed conserved synteny with the narrow-leafed lupin linkage group containing the equivalent *Ku* locus, which is controlled by an *FT* homologue (Nelson et al. 2017; Taylor et al. 2019).

### Vernalisation response in yellow lupin

A varying vernalisation response was observed among the parents and RIL population. The vernalisation response was moderate in domesticated parent Wodjil (accelerating flowering by 14 days) compared to very strong in the wild parent P28213 (accelerating flowering by 27 days). These findings suggest that vernalisation accelerates flowering in both wild and domesticated germplasm but to different degrees. The yellow lupin domesticated × wild RIL population shows less extreme variation in vernalisation responsiveness compared to the narrow-leafed lupin wild × domesticated RIL population, where the earlier parent has no vernalisation response (Nelson et al. 2017). Nevertheless, the bimodal distribution in the yellow lupin RIL population distribution (Fig. 2) allowed separation into two contrasting response types: high and low, which segregated in a 1:1 ratio and were mapped to a discrete Mendelian locus on linkage group YL-21 (Fig. 4). The mapping to one locus confirmed the one-gene control of this trait in yellow lupin rather than the alternate gene model of duplicate recessive gene action.

Despite the less extreme variation in vernalisation response in the yellow lupin RIL population than narrow-leafed lupin, the vernalisation response locus mapped to the same syntenic position as the vernalisation response locus (*Ku*) in narrow-leafed lupin (Fig. 6). This early *Ku* allele in the narrow-leafed lupin genome is derived by a spontaneous 1.4 kb deletion in the 5′ regulatory region of *LanFTc1*, one of four *FT* homologues (Nelson et al. 2017). The deletion appears to have derepressed the expression of *LanFTc1* in *Ku* types. Taylor et al. (2019) recently reported a second deletion allele at *LanFTc1*, which gives an intermediate vernalisation response. It would be fascinating to explore if a similar mutation has reduced vernalisation responsiveness in yellow lupin also. Rychel et al. (2019) recently reported a similar comparative analysis between white lupin and narrow-leafed lupin, although this was rather complex due to multigenic control of vernalisation response in white lupin. Thus, yellow lupin may serve as a simpler model for comparative analysis of vernalisation response in lupins.

A greater understanding of the molecular control of vernalisation response in yellow lupin would also open avenues of research to explore species-wide variation for phenological variation. Like other Mediterranean legumes, the strong vernalisation response in wild yellow lupin germplasm effectively adapts the plant to the areas with freezing winter temperatures where the crop originated. It protects wild genotypes from frost damage by keeping them in the frost tolerant vegetative phase of growth until a strong cool temperature influx triggers the transition to the more frost-sensitive flowering phase. Similarly, a moderate vernalisation requirement allows the yellow lupin crop to flower late in long growth environments with abundant moisture supply throughout the growing season and enables them to maximise vegetative growth and resource capture, ultimately resulting in higher seed yield. A weak level of vernalisation is beneficial in shorter season growth environments with terminal water-deficit conditions, where it allows plants to flower earlier and complete their life cycle earlier in order to escape the damaging effects of terminal drought (Berger et al. 2012; Berger and Ludwig 2014). This phenomenon of drought escape was clearly exhibited by this RIL population as it was revealed by time to maturity that all the genotypes under both treatments completed their life cycle at same time as the temperature rose above 25 °C to create terminal water-deficit-type conditions (Table S4). The temperatures above 25 °C result in flower and seed loss in lupins, thus in the total yield reduction (Kelleher 2003).

In this population, we observed segregation distortion pointing towards a duplicate recessive gene action for alkaloid content. But we tentatively attribute this segregation distortion to selection for bitter types as a result of inadvertent insect herbivory during RIL development in which 98 lines were lost between F_2_ and F_9_ (Iqbal et al. 2019). Nevertheless, the segregation distortion did not prevent accurate mapping. The same patterns appeared during the phenotypic and molecular characterisation of this trait in narrow-leafed lupin where the recessive *iucundus* allele confers sweetness or low alkaloid content in domesticated types (Gladstones 1977; Nelson et al. 2006). Recently, Kroc et al. (2019) identified an APETALA2/ethylene response transcription factor gene, *RAP2*-*7*, as a strong candidate gene for *iucundus*. However, since alkaloid content in yellow lupin maps to a different syntenic region in narrow-leafed lupin (chromosome NLL-20 rather than NLL-07 where *iucundus* is located), in yellow lupin alkaloid content is likely conferred by mutation in a different gene. Early efforts to develop low alkaloid narrow-leafed lupin and yellow lupin lupins identified three independent low alkaloid mutant genes: *dulcus*, *amoenus* and *liber* (Hackbarth and Sengbusch 1934; Hackbarth and Troll 1941). As it is unclear which (if any) of these low alkaloid mutants were incorporated into Wodjil, a selection from the Polish variety ‘Teo’, research is still required to determine the molecular mechanism of low alkaloid content in yellow lupin.

Flower and seed colour are important morphological markers in maintaining the integrity of genotypes through the identification of heterozygosity or mixing. The yellow lupin RIL population segregated in the expected 1:1 ratio for both flower and seed colour independently. The linkage mapping of each of these traits to one major locus confirmed the single-gene control of these traits in yellow lupin and not the duplicate recessive gene action model. By contrast, in narrow-leafed lupin flower and seed colour are both controlled by the single *leucospermus* locus (Nelson et al. 2006, 2010b). The wild narrow-leafed lupin has blue flowers and dark colour seeds, while domesticated narrow-leafed lupin has white flowers and white seeds (Nelson et al. 2006). The genome comparison of yellow lupin with narrow-leafed lupin and white lupin did not show any conserved synteny for flower and seed colour—unsurprising perhaps given their contrasting colours.

Phenotyping the RIL population showed that indehiscence, plant growth habit and seed permeability are each controlled by two loci. In narrow-leafed lupin, pod indehiscence is also under two-gene control (*tardus* and *lentus*) (Gladstones 1967). Unlike the two-gene control for seed permeability in yellow lupin, in narrow-leafed lupin seed permeability is controlled by the single recessive gene, *mollis* (Mikolajczyk 1966). The genetic control of growth habit has not been reported in narrow-leafed lupin. In chickpea, plant growth habit is controlled by a single gene with prostrate/rosette type growth habit dominant over erect/upright (Aryamanesh et al. 2010). The rosette type habit could be beneficial to retain soil moisture for extended periods through reducing evaporation of moisture by improved soil cover and competitive ability with weeds for available resources. By contrast, an erect/upright crop architecture aids agronomic management and harvesting. Yellow lupin types with intermediate growth habit may combine advantages of both extremes.

The map used is incomplete (Iqbal et al. 2019), as indicated by the excess of linkage groups (40 linkage groups rather than the target of 26 representing 26 chromosome pairs). This may be a factor in the lack of QTLs detected for time to maturity or length of reproductive phase (Fig. 4). Future mapping efforts should focus on alternative marker technologies such as targeted amplicon sequencing using transcriptome resources (unpublished data) for primer design.

### Genome-wide comparison between yellow lupin and narrow-leafed lupin

This is the first genome-wide comparison of three lupin genomes, building on a previous pairwise comparison between narrow-leafed lupin and white lupin (Hufnagel et al. 2020; Ksiazkiewicz et al. 2017). We found that yellow lupin has a more similar chromosome structure to white lupin than to narrow-leafed lupin consistent with their closer number of chromosomes, contrary to the supposed closer phylogenetic relationship between yellow lupin and narrow-leafed lupin (Naganowska et al. 2003). For example, most of the yellow lupin groups aligned to white lupin groups along more of their lengths than they did with narrow-leafed lupin groups. Additionally, there were more inversions and translocations in yellow lupin linkage groups compared to narrow-leafed lupin than comparing yellow lupin groups versus white lupin groups (Supplementary Table 2). The large number of chromosomal rearrangements between yellow lupin and narrow-leafed lupin may in part explain why crosses between these two species are difficult despite their apparent close phenotypic relationship (Kasten et al. 1991). However, progress has been made in crossing methodology that could potentially lead to the transfer of useful traits between these two species such as higher seed protein content and quality from yellow lupin into narrow-leafed lupin (Clements et al. 2009).

The sequence of chromosome rearrangement events remains unresolved so far. The picture will become clearer with current development of a reference genome for yellow lupin (J. Udall, pers. comm.) and improved reference genome for narrow-leafed lupin (K. Singh, pers. comm.). We are unaware of genetic mapping or reference genome development in other lupin species; however, transcriptome-based projects offer promise of improved resolution of lupin evolutionary history of New World (Nevado et al. 2016) and Old World (K. Susek, pers. comm.) lupins. The sequence tags available for GBS (100 bp) and DArTseq (69 bp) markers used to generate the yellow lupin map were too short to reliably detect orthologous sequences in the reference legume genome of *M. truncatula* (data not presented). A reference genome for yellow lupin would enable easier comparison to *M. truncatula* and other sequenced legume genomes, enabling more effective leveraging of functional genomic information generated in those model systems.

The reason for translocations observed at many genomic positions during comparative genomics of yellow lupin and narrow-leafed lupin may be because of cytogenetic differences among both species as yellow lupin contains 26 chromosome pairs (2*n* = 52) as compared to 20 chromosome pairs (2*n* = 40) in narrow-leafed lupin (Susek et al. 2016). These translocations were also observed between yellow lupin and white lupin, albeit to a lesser extent. The first 15 linkage groups of yellow lupin appear normal in that they each primarily align to one or two linkage groups of both narrow-leafed lupin (Fig. 5a, b), and they show a normal diagonal pattern (Supplementary Figure 2). The remaining yellow lupin groups seem to be mainly vertical indicating markers that have genetic recombination in yellow lupin but particularly, not in narrow-leafed lupin (Supplementary Figure 2). Two main conclusions could be drawn from this pattern: (1) we have marker clusters where the markers are likely physically close to each other but there are random (or systematic) genotyping errors which are creating a lot of apparent crossovers in yellow lupin, and (2) narrow-leafed lupin has many regions of suppressed recombination, whereas yellow lupin has abundant recombination in these areas. According to our overall experience with this mapping population, we consider that the former is much more likely because the narrow-leafed lupin and white lupin maps are so much better understood and more complete than this first draft of a yellow lupin map. Overall, despite yellow lupin map being less complete than the white lupin and narrow-leafed lupin maps, it provides a significant insight about the genetic control of key domestication traits in yellow lupin. The markers linked to key traits such as vernalisation response could be reliably utilised in marker-assisted selection for this trait in other species.

## Conclusion

This study of a cross between wild and domestic yellow lupins indicates simple (one gene) control of key domestication traits—flowering regulation through vernalisation, flower and seed colour, and alkaloid content, which opens the ways to tap the wild diversity in order to broaden genetic base by marker-assisted selection of these simply inherited key domestication traits. A more complex (two-gene) mechanism controls the traits pod dehiscence, seed permeability and plant growth habit. The genomic comparison of yellow lupin with narrow-leafed lupin and white lupin provides an avenue to further investigate the evolution of these sister species. The markers developed in the present study may be utilised for the marker-assisted breeding in yellow lupin.

## Electronic supplementary material

Below is the link to the electronic supplementary material.

Supplementary material 1 (DOCX 39 kb)

Supplementary material 2 (DOCX 159 kb)

Supplementary material 3 (XLSX 17 kb)

Supplementary material 4 (XLSX 14 kb)

Supplementary material 5 (XLSX 391 kb)

Supplementary material 6 (XLSX 17 kb)
