# Supplementary material for: Phenotypic characterisation and linkage mapping of domestication syndrome traits in yellow lupin (Lupinus luteus L.)
Source: Theor Appl Genet. 2020 Jul 18;133(10):2975–87. doi: 10.1007/s00122-020-03650-9 (PMC7497344; doi:10.1007/s00122-020-03650-9)
Supplement: Supplementary file 1 — Supplementary material 1 (DOCX 39 kb) [file 122_2020_3650_MOESM1_ESM.docx]

**Supplementary Fig 1 a)** Relationship between time to 50% flowering (x-axis) and reproductive phase (y-axis) under both (vernalised vs non-vernalised) treatments. **b)** Relationship between time to 50% flowering (x-axis) and time to maturity (y-axis) under both (vernalised vs non-vernalised) treatments

**a)**

**
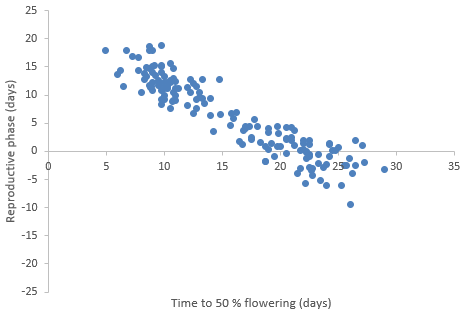
**

**b)**

**
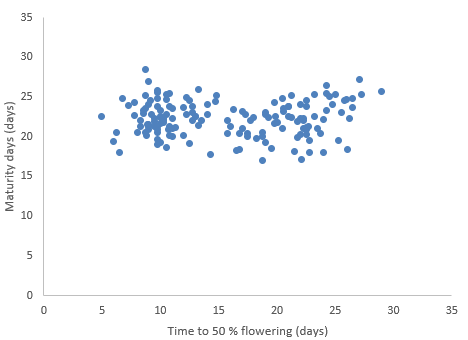
**
