# Supplementary material for: Phenotypic characterisation and linkage mapping of domestication syndrome traits in yellow lupin (Lupinus luteus L.)
Source: Theor Appl Genet. 2020 Jul 18;133(10):2975–87. doi: 10.1007/s00122-020-03650-9 (PMC7497344; doi:10.1007/s00122-020-03650-9)

##### **Supplementary Figure 2** Global distribution of synteny between 38 linkage groups of *L. luteus* (YL-01 to YL-38) and 20 chromosomes of *L. angustifolius* (NLL01 to NLL20). Loci showing homology between the two genomes at P < 1e-5 significance threshold are indicated by dots


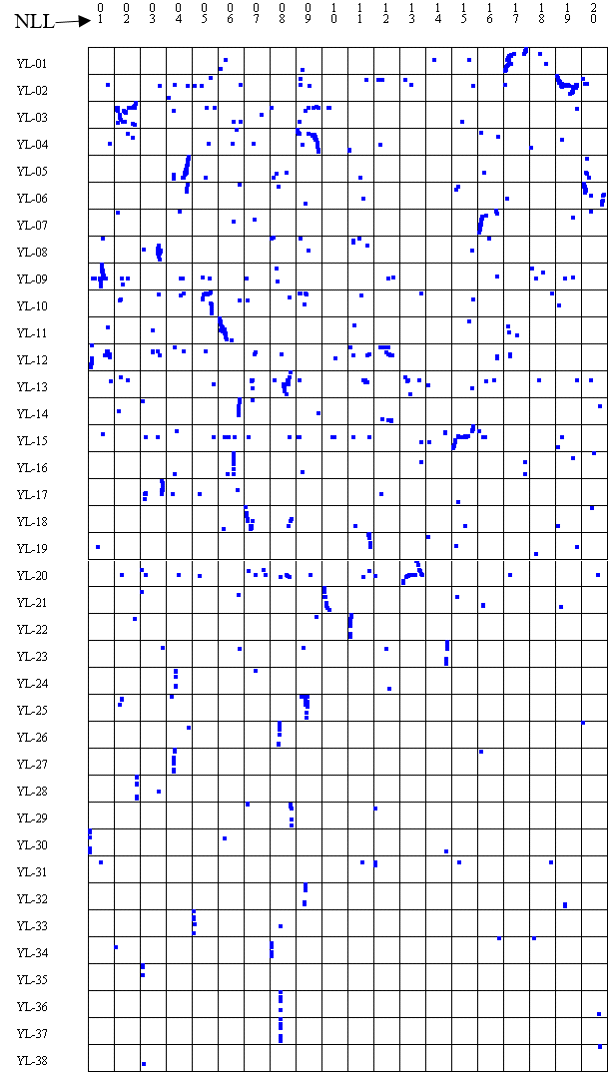

Supplement: Supplementary file 2 — Supplementary material 2 (DOCX 159 kb) [file 122_2020_3650_MOESM2_ESM.docx]
